# The Influence of Comorbidity on Health-Related Quality of Life After Esophageal Cancer Surgery

**DOI:** 10.1245/s10434-020-08303-1

**Published:** 2020-03-11

**Authors:** Lovisa Backemar, Asif Johar, Anna Wikman, Janine Zylstra, James Gossage, Andrew Davies, Jesper Lagergren, Pernilla Lagergren

**Affiliations:** 1 Surgical Care Science, Department of Molecular Medicine and Surgery, Karolinska Institutet, Karolinska University Hospital, Stockholm, Sweden; 2 Reproductive Health, Department of Women’s and Children’s Health, Uppsala University, Uppsala, Sweden; 3 Upper Gastrointestinal Surgery, Department of Molecular Medicine and Surgery, Karolinska Institutet, Karolinska University Hospital, Stockholm, Sweden; 4 School of Cancer and Pharmaceutical Sciences, Kings College, London, UK; 5 Upper Gastrointestinal Surgery, Guy’s and St Thomas’ NHS Trust, London, UK

## Abstract

**Background:**

Esophageal cancer surgery reduces patients’ health-related quality of life (HRQoL). This study examined whether comorbidities influence HRQoL in these patients.

**Methods:**

This prospective cohort study included esophageal cancer patients having undergone curatively intended esophagectomy at St Thomas’ Hospital London in 2011–2015. Clinical data were collected from patient reports and medical records. Well-validated cancer-specific and esophageal cancer-specific questionnaires (EORTC QLQ-C30 and QLQ-OG25) were used to assess HRQoL before and 6 months after esophagectomy. Number of comorbidities, American Society of Anesthesiologists physical status classification (ASA), and specific comorbidities were analyzed in relation to HRQoL aspects using multivariable linear regression models. Mean score differences with 95% confidence intervals were adjusted for potential confounders.

**Results:**

Among 136 patients, those with three or more comorbidities at the time of surgery had poorer global quality of life and physical function and more fatigue compared with those with no comorbidity. Patients with ASA III–IV reported more problems with the above HRQoL aspects and worse social function and pain compared with those with ASA I–II. Cardiac comorbidity was associated with worse global quality of life and dyspnea, while pulmonary comorbidities were related to coughing. Patients assessed both before and 6 months after surgery (*n* = 80) deteriorated in most HRQoL aspects regardless of comorbidity status, but patients with several comorbidities had worse physical function and fatigue and more trouble with coughing compared with those with fewer comorbidities.

**Conclusion:**

Comorbidity appears to negatively influence HRQoL before esophagectomy, but appears not to severely impact 6-month recovery of HRQoL.

Globally, esophageal cancer is common and carries a poor prognosis (overall 5-year survival of < 20%) and is thus the sixth most common cause of cancer death.^1^,^2^ The most established curatively intended treatment involves esophagectomy, often combined with neoadjuvant treatment.

Mortality and complication rates after esophagectomy have decreased during recent years, probably at least partly explained by the centralization of services, development of surgical techniques, and improvements in perioperative care^3^^–^5; however, the postoperative 5-year survival rate is still only 30–40%.^6^ The extensive nature of esophagectomy greatly reduces patients’ health-related quality of life (HRQoL),^7^^–^9 which is a crucial aspect of survivorship for these patients.^10^,^11^ There seems to be limited improvement in HRQoL from 6 months to 5 years after surgery.^7^,^9^,^12^

Comorbidity is a factor that might influence patients’ recovery of HRQoL after esophagectomy.^13^,^14^ Comorbidities are considered when evaluating whether a patient is a surgical candidate or not. Comorbidities affect HRQoL in the short term after esophageal cancer surgery,^15^ while in the longer term it has been shown that preoperative comorbidities are associated with poorer global quality of life and more problems with fatigue and dyspnea.^13^ However, better knowledge of how comorbidities affect HRQoL after esophagectomy can provide value for clinical decision making, which in turn can help reduce suffering and improve the life situation, and even survival, among patients.

The purpose of this study was therefore to examine the influence of comorbidities on HRQoL at the time of surgery, and change in HRQoL until 6 months postoperatively.

## Methods

### Design

This was a prospective, single-center cohort study of esophageal cancer patients who underwent curatively intended esophagectomy at St Thomas’ Hospital, London, UK. Patient recruitment commenced on 1 November 2011 and ended on 28 February 2015. For the 6-month analysis, patients were included until 31 August 2014 and the 6-month follow-up ended on 28 February 2015. Patients were identified by both the surgical team and the study coordinators during clinical consultations, at presentation in multidisciplinary case meetings, or following screening of medical records. Detailed information about patient characteristics, including weight and height, treatment, and predefined comorbidities was prospectively collected on the basis of a detailed and predefined study protocol. The American Society of Anesthesiologists classification for physical status score (ASA) was assigned by the anesthesiologist when structurally reviewing the patient’s physical status at the time of esophagectomy. HRQoL data were collected before surgery and 6 months after surgery using self-report questionnaires developed and validated by the European Organization for Research and Treatment of Cancer (EORTC).^16^

### Comorbidity Exposure

Exposures were the number of comorbidities, ASA classification, and specific comorbidities. The ASA classification is a readily available and widely accepted system used before surgical procedures to assess the physical fitness of a patient on the basis of their comorbidities. It was first introduced in 1941 and has been developed into a six-category physical status system:^17^,^18^ ASA I, a healthy patient; ASA II, a patient with mild systemic disease (e.g. well-controlled diabetes mellitus or hypertension, mild lung disease, and cancer); ASA III, a patient with severe systemic disease (e.g. poorly controlled diabetes mellitus, chronic obstructive pulmonary disease [COPD], morbid obesity, or history of myocardial infarction); ASA IV, a patient with severe systemic disease that is a constant threat to life (e.g. ongoing cardiac ischemia, severe valve dysfunction, or sepsis); ASA V, a moribund patient who is not expected to survive without the operation; and ASA VI, a declared brain-dead patient whose organs are being removed for donor purposes.^19^ It is a partly subjective assessment, made by the anesthesiologist prior to surgery, but it is a well-established system that has been shown to be a reliable way of classifying preoperative physical status.^20^,^21^ For the purposes of the present study, patients were categorized into two ASA categories: ASA I–II, representing otherwise healthy patients, and ASA III–IV, representing patients with substantial comorbidity. However, in practice, these categories represented ASA II and ASA III, respectively, because no ASA I patients were included since all had a cancer diagnosis, there were very few ASA IV patients, and patients in ASA groups V–VI were never considered for surgery.

The number of significant and predefined comorbidities were also analyzed and patients were categorized as having: (1) no comorbidity; (2) one comorbidity; (3) two comorbidities; or (4) three or more comorbidities. Comorbidities included in the variable ‘number of comorbidities’ were the same as the specific comorbidities described below.

Finally, the following specific comorbidities assessed before surgery were analyzed separately: (1) cardiac disease (including angina, previous myocardial infarction, and heart failure); (2) hypertension (requiring medication); (3) diabetes; (4) pulmonary disease (COPD and asthma); (5) obesity (body mass index ≥ 30 just before surgery); and (6) other comorbidities (used only for adjustment in the statistical model, including other [non-esophageal] cancers, cerebrovascular disease, kidney failure, and mental disorders, including depression, rheumatoid arthritis or other significant diseases, defined by experienced physicians while manually reviewing the medical charts).

### Health-Related Quality-of-Life Outcomes

HRQoL was assessed by the EORTC questionnaires QLQ-C30 and QLQ-OG25 before surgery and 6 months postoperatively. The QLQ-C30 is a general cancer-related HRQoL questionnaire that incorporates 30 questions distributed on nine multi-item scales measuring functions (global quality of life, physical, role, cognitive, emotional, and social functioning) and symptoms (fatigue, pain, nausea, and vomiting), and six single items measuring general symptoms (dyspnea, appetite loss, insomnia, constipation, diarrhea, and financial impact).^16^ The QLQ-OG25 is an esophagogastric-specific module that comprises 25 items distributed on six multi-item scales (dysphagia, eating restrictions, reflux, odynophagia, pain, and anxiety) and 10 single items (eating with others, dry mouth, trouble with taste, body image, trouble swallowing saliva, choking when swallowing, trouble with coughing, trouble talking, weight loss, and hair loss).^22^ Items were scored on a 4-point Likert-type scale: (1) not at all; (2) a little; (3) quite a bit; and (4) very much, except for the global quality-of-life scale, which was scored on a 7-point scale ranging from (1) very poor to (7) excellent. A priori, specific aspects of HRQoL were selected based on previous literature on how comorbidities affect HRQoL,^13^,^23^ and included global quality of life, physical function, emotional function, social function, fatigue, pain, and dyspnea from the QLQ-C30; and reflux, anxiety, and trouble with coughing from the QLQ-OG25. The remaining aspects were not analyzed in order to reduce multiple testing errors.

### Statistical Analysis

Responses were transformed linearly to a 0- to 100-point scale according to the EORTC scoring manual, and missing responses were handled accordingly.^24^ High scores on the global quality-of-life and function scales correspond to better HRQoL, while higher scores on symptom scales and single items reflect worse symptoms and poorer HRQoL. Mean score differences (MDs) were calculated between exposure groups by subtracting the exposure group score from the reference category score, and over time by subtracting the preoperative score from the score at 6 months follow-up. Linear regression models were used to assess exposure groups in relation to MDs with 95% confidence intervals (CIs) for the selected HRQoL scales and items. HRQoL scores before surgery were compared between patients’ number of comorbidities (0 = reference, 1, 2, or ≥ 3), ASA scores (I–II = reference, III, or IV), and specific comorbidities (absence of specific comorbidity = reference, cardiac disease, hypertension, diabetes, pulmonary disease, or obesity). Comparisons of the HRQoL scores 6 months after surgery were analyzed regarding the number of comorbidities and ASA score groups. Linear regression analysis further examined changes in HRQoL scores over time between the assessments before surgery and 6 months after surgery. Only the number of comorbidities and ASA score groups were assessed, since the specific comorbidities were too infrequent to allow for a robust analysis. Based on previous research, an MD between assessments or between exposure groups of ≥ 10 was considered clinically relevant.^25^ For all models, adjustments were made for potential confounding by age (categorized into < 65 years or ≥ 65 years), sex (male or female), tumor stage (0–II or III–IV), tumor histology (squamous cell carcinoma or adenocarcinoma), neoadjuvant treatment (yes or no), surgical approach (transhiatal esophagectomy, thoracoabdominal esophagectomy, or laparoscopic esophagectomy), postoperative complications (yes or no, regarding predefined complications occurring within 30 days of surgery), tobacco smoking status (non-smoker or current smoker at the time of surgery) and other comorbidities (all comorbidities were included except the one being analyzed). To increase the power in the regression models, missing values were used as separate categories since a sensitivity analysis comparing these models with models excluding all patients with missing data showed similar results.

### Ethical Considerations

At a presurgery hospital visit or admission to hospital, patients were invited to participate, at which point full study information was provided and written informed consent obtained. Ethical approval was granted by the National Research Ethics Service Committee London—London Bridge (REC reference 11/LO/0335) and the National Research Ethics Service, West Midlands (REC reference 13/WM/0131).

## Results

### Patients

Among 171 patients who underwent esophagectomy for esophageal cancer at Thomas’ Hospital, London, UK, during the study period, and who were thus eligible for the study, 12 (7%) declined participation, 18 (11%) did not complete the questionnaires, and 5 (3%) had missing information on tobacco smoking (4 patients) or tumor stage (1 patient), leaving 136 (80%) patients for the presurgery analysis. At 6 months after surgery, 103 patients were eligible to participate, among whom 23 (22%) did not complete the questionnaires or had died, leaving 80 (78%) patients for final analysis of changes in HRQoL between the period before and 6 months after surgery. Characteristics of the responders and non-responders to the questionnaires were similar, although complications were less common among non-responders (25% vs. 45%). Compared with the patients who responded to the follow-up questionnaire, patients who did not respond had similar baseline values in all aspects, except for a stronger male predominance (96% vs. 74%) and poorer global quality of life (MD −  14, 95% CI −  25 to −  3). Characteristics of the patients who participated in the presurgery assessment are presented in Table 1. Most patients were male (79%), had adenocarcinoma (90%), and received neoadjuvant therapy (87%). Characteristics were generally equally common among patients with different numbers of comorbidities; however, patients aged ≥ 65 years had more comorbidities.

**Table 1:** Patient and tumor characteristics of 136 study patients with esophageal cancer who underwent surgery

	Total [*n *= 136]		No comorbidity [*n *= 44]		One comorbidity [*n *= 39]		Two comorbidities [*n *= 34]		Three or more comorbidities [*n *= 19]		ASA I–II [*n *= 97]		ASA III–IV [*n *= 39]	
	*n*	%	*n*	%	*n*	%	*n*	%	*n*	%	*n*	%	*n*	%
*Age, years*														
< 65	63	46	22	50	19	49	14	41	8	42	45	46	18	46
≥ 65	73	54	22	50	20	51	20	59	11	58	52	54	21	54
*Sex*														
Male	108	79	33	75	32	82	28	82	15	79	75	77	33	85
Female	28	21	11	25	7	18	6	18	4	21	22	23	6	15
*Tumor stage*^I^														
0–II	43	32	18	41	11	28	10	29	4	21	30	31	13	33
III–IV	93	68	26	59	28	72	24	71	15	79	67	69	26	67
*Tumor histology*														
Adenocarcinoma	123	90	41	93	33	85	31	91	18	95	86	89	37	95
Squamous cell carcinoma	13	10	3	7	6	15	3	9	1	5	11	11	2	5
*Neoadjuvant therapy*^I^														
Yes	118	87	36	82	35	90	29	85	18	95	85	88	33	85
No	18	13	8	18	4	10	5	15	1	5	12	12	6	15
*Surgical approach*														
Transhiatal	74	54	25	57	19	49	17	50	13	68	51	53	23	59
Thoracoabdominal	45	33	14	32	16	41	10	29	5	26	34	35	11	28
Laparoscopic	17	13	5	11	4	10	7	21	1	5	12	12	5	13
*Complications*														
Yes	53	39	16	36	15	38	14	41	8	42	38	39	15	38
No	83	61	28	64	24	62	20	59	11	58	59	61	24	62
*Tobacco smoking*														
Yes	58	43	18	41	19	49	12	35	9	47	45	46	13	33
No	78	57	26	59	20	51	22	65	10	53	52	54	26	67

^I^Missing values of covariates were missing at random and were considered as separate groups

*ASA* American Society of Anesthesiologists physical status classification

### Presurgical Health-Related Quality of Life

Comorbidity was associated with poorer HRQoL before surgery, on several aspects. Patients with three or more comorbidities had clinically and statistically significantly poorer global quality of life (MD −  14, 95% CI −  27 to −  2), worse physical function (MD −  12, 95% CI −  22 to −  2), and more fatigue (MD 18, 95% CI 6–31) compared with patients without comorbidity. However, comorbidities were not associated with poorer HRQoL on emotional function, reflux, or trouble with coughing (Table 2).

**Table 2:** Comorbidities, ASA, and HRQoL before surgery in esophageal cancer in 136 study patients

	One comorbidity		Two comorbidities		Three or more comorbidities		ASA III–IV	
	MD^II^	95% CI	MD^II^	95% CI	MD^II^	95% CI	MD^III^	95% CI
*QLQ*-*C30*								
Global quality of life	− 10	(− 20, 0)	− 7	(− 17, 3)	− 14	(− 27, − 2)	− 11	(− 19, − 2)
Physical function	− 11	(− 19, − 3)	− 1	(− 10, 7)	− 12	(− 22, − 2)	− 16	(− 23, − 10)
Emotional function	− 1	(− 11, 10)	9	(− 2, 19)	− 1	(− 14, 12)	− 6	(− 14, 3)
Social function	− 9	(− 22, 3)	1	(− 12, 13)	− 7	(− 22, 9)	− 13	(− 23, − 2)
Fatigue	16	(6, 26)	7	(− 4, 17)	18	(6, 31)	15	(6, 23)
Pain	7	(− 3, 18)	5	(− 7, 16)	7	(− 7, 21)	13	(4, 22)
Dyspnea	14	(3, 24)	0	(− 11, 10)	11	(− 3, 24)	8	(− 1, 18)
*QLQ*-*OG25*								
Reflux	1	(− 8, 11)	4	(− 6, 14)	0	(− 12, 12)	6	(− 2, 14)
Anxiety	− 8	(− 21, 5)	− 12	(− 26, 1)	7	(− 9, 23)	− 2	(− 13, 9)
Trouble with coughing	3	(− 7, 12)	− 1	(− 10, 9)	4	(− 8, 16)	3	(− 5, 11)

Data are expressed as adjusted^I^ MDs with 95% CIs

*ASA* American Society of Anesthesiologists physical status classification, *HRQoL* health-related quality of life, *MDs* mean score differences, *CIs* confidence intervals

^I^Adjusted for age, sex, tumor stage, histology of the tumor, neoadjuvant treatment, operation type, postoperative complications, tobacco smoking, and other comorbidities (including all comorbidities except the one being analyzed)

^II^No comorbidity was used as the reference category

^III^ASA score I–II was used as the reference category

Compared with patients with an ASA score of I–II, patients with an ASA score of III–IV experienced worse global quality of life (MD −  11, 95% CI − 19 to − 2), worse physical function (MD − 16, 95% CI − 23 to − 10), worse social function (MD − 13, 95% CI − 23 to − 2), more problems with fatigue (MD 15, 95% CI 6–23), and more pain (MD 13, 95% CI 4–22).

For specific comorbidities, patients with cardiac disease had clinically and statistically significantly worse global quality of life (MD − 15, 95% CI − 26 to − 3) and more dyspnea (MD 17, 95% CI 5–29) compared with those without these comorbidities. A clinically relevant difference (MD ≥ 10) was also seen for social function, fatigue, pain, and anxiety for such patients but these MDs were not statistically significant (Table 3). Patients with pulmonary comorbidities experienced clinically, but not statistically, significantly more trouble with coughing (MD 11, 95% CI 0–22) and more dyspnea (MD 10, 95% CI − 2 to 22). Patients with diabetes, hypertension, and obesity did not report poorer HRQoL on any of the aspects, except worse social function among patients with diabetes (MD 14, 95% CI 0 − 28) [Table 3].

**Table 3:** Specific comorbidities and HRQoL before surgery for esophageal cancer in 136 study patients

	Cardiac disease		Hypertension		Pulmonary disease		Diabetes		Obesity	
	MD^II^	95% CI	MD^II^	95% C)	MD^II^	95% CI	MD^II^	95% CI	MD^II^	95% CI
*QLQ*-*C30*										
Global quality of life	− 15	− 26, − 3	2	− 7, 11	− 9	− 20, 3	− 2	− 13, 9	5	− 5, 14
Physical function	− 9	− 19, 1	0	− 7, 8	− 2	− 12, 8	0	− 10, 9	4	− 4, 11
Emotional function	− 9	− 21, 3	− 2	− 11, 7	7	− 5, 19	3	− 9, 15	8	− 2, 17
Social function	− 11	− 26, 3	− 1	− 12, 10	− 4	− 19, 10	14	0, 28	5	− 7, 16
Fatigue	13	0, 25	4	− 6, 13	5	− 8, 17	− 4	− 16, 8	− 1	− 11, 8
Pain	12	− 2, 24	2	− 8, 11	− 4	− 17, 9	− 5	− 17, 8	− 5	− 16, 5
Dyspnea	17	5, 29	0	− 9, 9	10	− 2, 22	− 8	− 20, 4	− 8	− 18, 1
*QLQ*-*OG25*										
Reflux	9	− 3, 20	− 4	− 13, 5	6	− 5, 17	0	− 11, 12	− 5	(− 14, 4)
Anxiety	15	0, 31	− 4	− 15, 8	5	− 10, 21	0	− 15, 15	− 9	(− 21, 4)
Trouble with coughing	− 2	− 14, 9	− 4	− 13, 4	11	0, 22	2	− 9, 13	− 1	(− 10, 8)

Data are expressed as adjusted^I^ MDs with 95% CIs

*HRQoL* health-related quality of life, *MDs* mean score differences, *CIs* confidence intervals

^I^Adjusted for age, sex, tumor stage, histology of the tumor, neoadjuvant treatment, operation type, postoperative complications, tobacco smoking, and other comorbidities (including all comorbidities except the one being analyzed)

^II^The absence of that specific comorbidity was used as the reference category

### Changes in Health-Related Quality of Life from Presurgery to 6 Months Postoperatively

All patients deteriorated in several aspects of HRQoL during the period before and 6 months after surgery (Table 4). For global quality of life, patients without comorbidity had the greatest deterioration (MD − 22, 95% CI − 38 to − 8). For patients in the comorbidity groups, there were clinically, but not statistically, significant deteriorations in global quality of life for patients with two comorbidities (MD − 12) and three or more comorbidities (MD − 14). Patients with comorbidities had poorer global quality of life before surgery (i.e. worse baseline scores), therefore there was no difference in mean scores 6 months after surgery between patients with or without comorbidities. For pain and reflux, both the no comorbidity group and the comorbidity groups had clinically and statistically significant deterioration between the period before and 6 months after surgery, but mean scores at 6 months were similar, although starting from different levels (Table 4). For physical function, patients with no comorbidity, two comorbidities, and three or more comorbidities all deteriorated (MD − 18, 95% CI − 32 to − 5; MD − 22, 95% CI − 34 to − 10; MD − 18, 95% CI − 34 to − 3, respectively). Similar results were found for fatigue, and patients with three or more comorbidities also had a clinically relevantly worse mean score at 6 months compared with patients with no comorbidity (mean score 62 vs. 43). For trouble with coughing, patients with two and three or more comorbidities deteriorated (MD 25, 95% CI 9–40; MD 16, 95% CI clinically relevantly worse mean 4–35, respectively), and the mean scores 6 months after surgery were statistically significantly worse compared with patients with no comorbidity and one comorbidity. No changes in emotional function and anxiety were observed over time (Table 4).

**Table 4:** Comorbidities and differences in HRQoL between presurgical scores and scores 6 months after surgery, as well as mean scores at 6 months after surgery, for esophageal cancer in 80 study patients

	No comorbidity			One comorbidity			Two comorbidities			Three or more comorbidities		
	6 months after versus before surgery		Mean scores at 6 months	6 months after versus before surgery		Mean scores at 6 months	6 months after versus before surgery		Mean scores at 6 months	6 months after versus before surgery		Mean scores at 6 months
	MD	95% CI		MD	95% CI		MD	95% CI		MD	95% CI	
*QLQ*-*C30*												
Global quality of life	− 22	− 38, − 8	53	− 8	− 24, 7	59	− 12	− 27, 2	60	− 14	− 32, 3	45
Physical function	− 18	− 32, − 5	70	− 9	− 23, 5	66	− 22	− 34, − 10	67	− 18	− 34, − 3	60
Emotional function	0	− 14, 13	64	5	− 9, 20	71	− 6	− 18, 7	76	− 2	− 19, 14	64
Social function	− 16	− 35, 3	56	− 12	− 32, 8	55	− 24	− 41, − 6	55	− 18	− 40, 5	50
Fatigue	28	14, 43	43	11	− 5, 27	48	27	13, 40	50	27	9, 44	62^II^
Pain	29	11, 47	36	26	7, 45	45	19	2, 35	30	23	2, 44	40
Dyspnea	15	− 2, 31	27	2	− 16, 19	25	13	− 2, 28	21	17	− 3, 37	37
*QLQ*-*OG25*												
Reflux	31	11, 50	44	33	12, 54	45	30	12, 49	45	36	12, 59	47
Anxiety	− 6	− 25, 12	71	− 3	− 22, 16	58	− 7	− 24, 10	51^II^	− 5	− 26, 16	75
Trouble with coughing	2	− 14, 19	19	2	− 15, 19	24	25	9, 40	36^II^	16	− 4, 35	34

Data are expressed as adjusted^I^ MDs with 95% CIs and mean scores

*HRQoL* health-related quality of life, *MDs* mean score differences, *CIs* confidence intervals

^I^Adjusted for age, sex, tumor stage, histology of the tumor, neoadjuvant treatment, operation type, postoperative complications, and tobacco smoking

^II^Statistical significant (*p* < 0.05) and  clinically relevant (score difference ≥ 10) compared with means score of patients with no comorbidities

Patients with ASA I–II had similar deterioration in most HRQoL aspects as those with ASA III–IV (Table 5). For example, for global quality of life, both groups had lower scores (MD − 15, 95% CI − 28 to − 2; and MD − 13, 95% CI − 29 to 2, respectively) and experienced similar mean scores at 6 months after surgery. For physical function, social function, and fatigue, both groups deteriorated, but patients with ASA III–IV had either clinically relevant or statistically significantly worse mean scores 6 months after surgery than ASA I–II (Table 5).

**Table 5:** ASA scores and differences in HRQoL between presurgical scores and scores 6 months after surgery, as well as mean scores at 6 months after surgery, for esophageal cancer in 80 study patients

	ASA I–II			ASA III–IV		
	6 months after versus before surgery		Mean scores	6 months after versus before surgery		Mean scores
	MD	95% CI		MD	95% CI	
*QLQ*-*C30*						
Global quality of life	− 15	− 28, − 2	58	− 13	− 29, 2	54
Physical function	− 19	− 30, − 8	69	− 16	− 30, − 2	59
Emotional function	− 2	− 14, 9	73	− 0	− 14, 14	63
Social function	− 18	− 33, − 2	58	− 22	− 41, − 2	42^II^
Fatigue	23	13, 37	46	20	5, 36	59^II^
Pain	25	11, 40	34	17	− 1, 35	41
Dyspnea	11	− 3, 24	23	15	− 3, 34	32
*QLQ*-*OG25*						
Reflux	31	15, 47	44	33	13, 53	48
Anxiety	− 7	− 22, 7	60	0	− 18, 17	60
Trouble with coughing	12	− 2, 27	27	13	− 5, 31	34

Data are expressed as adjusted^I^ MDs with 95% CIs and mean scores

*ASA* American Society of Anesthesiologists physical status classification, *HRQoL* health-related quality of life, *MDs* mean score differences, *CIs* confidence intervals

^I^Adjusted for age, sex, tumor stage, histology of the tumor, neoadjuvant treatment, operation type, postoperative complications, and tobacco smoking

^II^Statistical significant (*p* < 0.05) and clinically relevant (score difference ≥ 10) compared with means score of patients with ASA I–II

## Discussion

This study showed that esophageal cancer patients with more comorbidities and higher ASA scores experience poorer HRQoL before surgery for esophageal cancer, particularly those with cardiac or pulmonary disease. However, most patients deteriorate in HRQoL to a similar extent regardless of comorbidity status and ASA score. However, patients with two or three or more comorbidities had worse physical function, fatigue, and trouble with coughing 6 months after surgery, and patients with ASA III–IV had worse physical function, social function, and fatigue than patients with ASA I–II. Regarding specific comorbidities, cardiac disease predisposed patients to worse global quality of life and dyspnea, and pulmonary disease predisposed patients to more trouble with coughing presurgery, while hypertension, diabetes, and obesity did not.

The methodological strengths of this study include its prospective design and the quality of the data obtained according to a predefined study protocol. In the statistical analysis, there was a selection of key outcomes from the EORTC questionnaires in order to reduce the risk of multiple testing errors. Moreover, the ability to adjust the results for several relevant confounding factors was advantageous. However, data on some potential confounders were not included, e.g. postoperative oncological therapy. In the adjustment for confounding by complications, we could not take severity of the complication into account, but studies identifying a strong association between complications and postoperative HRQoL have measured complications as present or not and thus this approach was considered acceptable as for adjustment.^26^,^27^ The high rate of non-responders at the 6-month follow-up increases the risk of selection bias. However, in a drop-out analysis, responders and non-responders were similar regarding most characteristics, hence strong selection bias seems unlikely. The preoperative assessment of HRQoL might be influenced by the cancer diagnosis, neoadjuvant treatment, and upcoming surgery. However, most patients recover their HRQoL after neoadjuvant treatment prior to surgery.^28^,^29^ Use of adjuvant treatment could have influenced the postoperative HRQoL outcomes. Additionally, patients with and without comorbidity were measured under the same circumstances, which should make the comparison valid. The lack of detailed information on the severity of comorbidities is a limitation. Patients selected for surgery, despite them having comorbidities, may, for example, have a better performance status than patients suffering from the same comorbidities who were not selected. However, the ASA classification has been developed to take the severity of the comorbidity into account by giving higher scores to more severe diseases, which has been well-validated to classify preoperative physical status.^20^,^21^

The results of the present study indicate that comorbidities, ASA classification, cardiac disease, and pulmonary disease affected some aspects of HRQoL among cancer patients before esophagectomy, but higher comorbidity status or ASA classification indicated worse deterioration of HRQoL for only a few aspects at 6 months after surgery. However, for most aspects, patients with comorbidities had worse HRQoL at baseline compared with patients without comorbidities. Then, at 6 months after surgery, patients without comorbidities worsened in most aspects, resulting in similar scores as patients with comorbidities. Patients with comorbidities may have adapted to a lower HRQoL and may not have been so greatly affected by surgery as patients without comorbidity. This was not the case for physical function, fatigue, and trouble with coughing, because patients with two or three or more comorbidities deteriorated from presurgery to 6 months and had worse scores at 6 months than patients without comorbidities. There is evidence that comorbidities in general affect HRQoL in the short term after esophageal cancer surgery,^15^ and in the longer term it has been seen that preoperative comorbidities are associated with worse scores for global quality of life, fatigue, and dyspnea in these patients.^13^ There is a need to investigate the impact of comorbidities in the longer term after surgery because new comorbidities diagnosed during follow-up and the recovery period in HRQoL could vary between groups of patients.

There are studies investigating how comorbidities influence HRQoL (using the SF-36 questionnaire) in other diagnoses, concluding that common chronic comorbidities (chronic lung disease, congestive heart failure, diabetes, hypertension, ischemic heart disease) have a negative impact on HRQoL in general.^30^,^31^ In patients undergoing surgery for prostate cancer, the number of preoperative comorbidities was associated with poorer HRQoL regarding physical and disease-specific aspects, but not mental aspects.^32^ It has also been shown that preoperative ASA class is a prognostic factor for quality of life 6 months after surgery in general.^33^,^34^ One other study using the EORTC QLQ-C30 found that HRQoL worsened if cancer survivors (all cancers) suffered from hypertension and diabetes.^35^

## Conclusions

This study indicates that the presence of comorbidities influences many preoperative HRQoL aspects, as well as recovery in physical function, fatigue, and coughing in esophageal cancer patients undergoing esophagectomy. In most other aspects, patients with and without comorbidities deteriorate to the same level of HRQoL 6 months after surgery. In addition, patients with a higher ASA score, cardiac disease, or pulmonary disease experience worse HRQoL in some aspects before surgery. It is important to be aware of the effect of comorbidity through the whole treatment processes this study assesses. Information on comorbidity is therefore of value, especially in a clinical setting, in order to inform patients and to guide tailored follow-up and interventions until long after treatment. Additionally, the knowledge of how comorbidities affect HRQoL may help to better tailor the treatment for these patients and improve their well-being. Patients with comorbidities might need more intervention and information before surgery, but 6 months after surgery most patients seem to be in need of actions to help improve their HRQoL irrespective of their comorbidity status.
